# Phytochemical Characterization, Antioxidant, Antimicrobial and Cytotoxic Activities of *Seseli transcaucasicum* (Schischk.) Pimenov & Sdobnina

**DOI:** 10.3390/plants15172584

**Published:** 2026-08-25

**Authors:** Mehmet Şirin Karan, Mustafa Yunus Emre

**Affiliations:** 1 Department of Biology, Mardin Artuklu University Graduate Education Institute, Mardin 47200, Turkey; mehmet.karan.59@gmail.com; 2 Nursing Department, Faculty of Health Sciences, Mardin Artuklu University, Mardin 47200, Turkey

**Keywords:** *Seseli transcaucasicum*, phenolic compounds, antioxidant activity, antimicrobial activity, cytotoxicity

## Abstract

Species belonging to the genus *Seseli* have long been known to be used as herbal remedies in traditional medicine for various purposes such as colds, inflammation, pain, and gastrointestinal disorders. Therefore, in this study, the flower parts of *Seseli transcaucasicum* (Schischk.) Pimenov & Sdobnina were extracted using methanol and acetone to investigate their phytochemical composition and antioxidant, antimicrobial, and cytotoxic activities. For this purpose, total phenolic content (TPC), DPPH free radical scavenging activity (RSA), phenolic component (PCs) profile, volatile component composition, antimicrobial effect, and cytotoxic potential on the HT29 human colorectal adenocarcinoma cell line were evaluated. When the results were examined, HPLC analysis of phenolic components revealed that both extracts contained particularly high levels of rosmarinic acid, catechin, and quercetin. Among these, rosmarinic acid was detected in the highest amount, with 15.195 mg/g in the methanol extract and 14.929 mg/g in the acetone extract. The volatile components of this plant were determined by GC-MS, and a total of 29 volatile compounds were identified. Among these, β-pinene (220.792 mg/kg), Δ3-carotene (81.898 mg/kg), α-sedrene (63.331 mg/kg), elixen (44.535 mg/kg), sabinene (40.822 mg/kg), and D-limonene (38.422 mg/kg) were identified as the most dominant compounds. It was observed that the methanol extract had a higher total phenolic content (77.389 ± 1.389 mg gallic acid equivalent/g extract) and higher free radical scavenging activity (40.933 ± 0.067 mg trolox equivalent/g extract) compared to the acetone extract. *Escherichia coli*, *Pseudomonas aeruginosa*, *Staphylococcus aureus*, and *Candida albicans* strains were used to determine the antimicrobial activities of plant extracts, and moderate inhibitory effects were observed against these microorganisms. The highest antimicrobial activity was determined by the 16 mm inhibition zone created against *S. aureus* by the methanol extract at a concentration of 100 mg/mL. The cytotoxic activity of the plant was determined by measuring its dose-dependent antiproliferative effect on HT29 cells. When the difference between solvents was examined, it was observed that the acetone extract at a concentration of 500 µg/mL reduced cell viability by 18.03%, while the methanol extract at the same concentration reduced viability by 30.56%. According to the results, *S. transcaucasicum* is rich in phenolic and terpenic compounds and exhibits antioxidant, antimicrobial, and cytotoxic effects. Therefore, this plant species is considered a promising resource for the development of naturally occurring biologically active compounds.

## 1. Introduction

*Seseli* species are perennial, aromatic herbaceous plants with woody main roots and erect stems belonging to the Apiaceae family. The most distinctive chemical feature of the genus is its high richness in pyranocoumarins and furanocoumarins. These secondary metabolites play a role in the plant′s defense system and are also noteworthy in the medical world due to their anti-inflammatory, spasmolytic and especially phototoxic effects [1,2].

**Figure 1:**
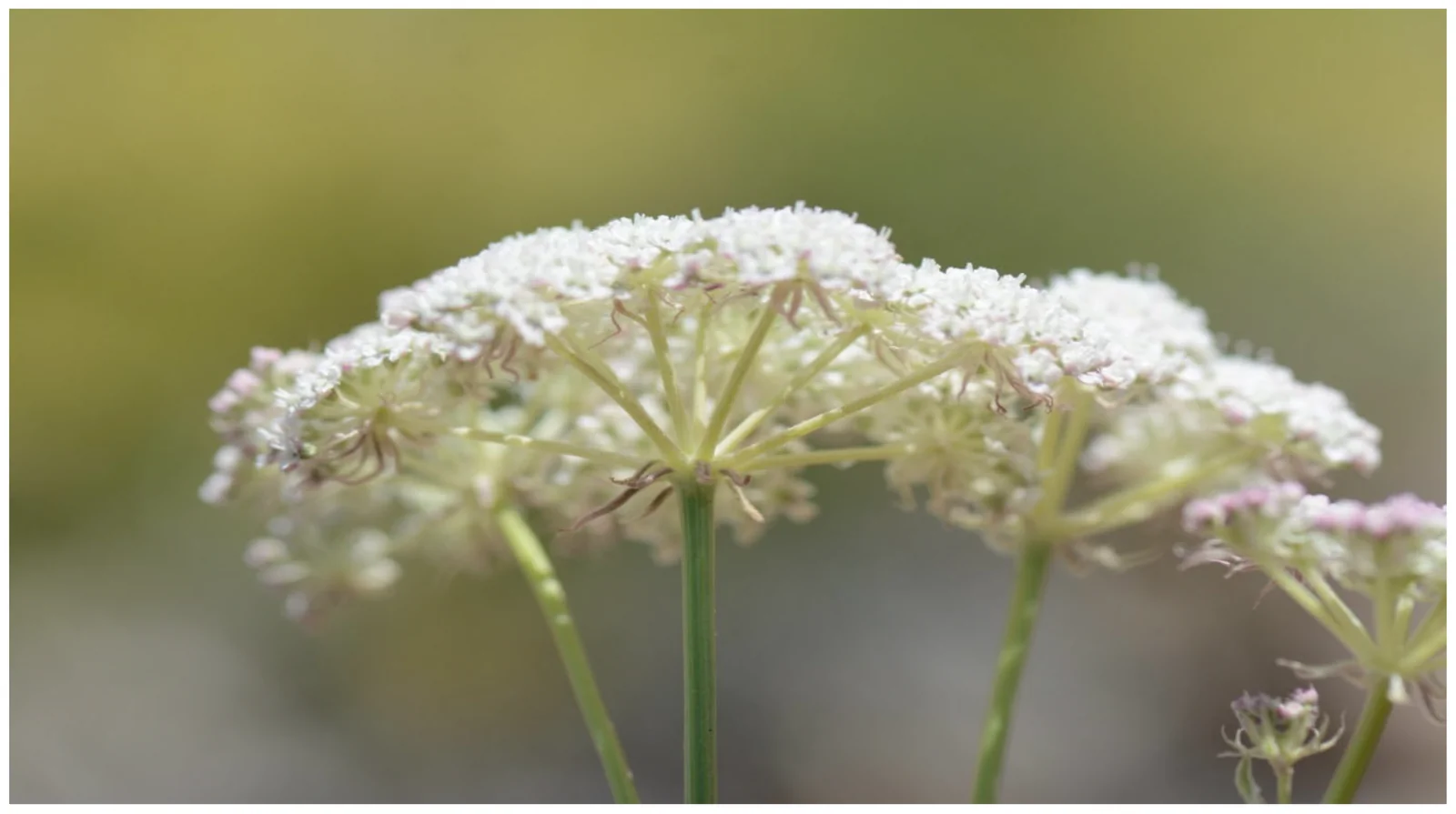
Image of the *Seseli transcaucasicum* plant used in the study.

*Seseli transcaucasicum* (Schischk.) Pimenov & Sdobnina is a species widely distributed in Türkiye, particularly in the Eastern Anatolia Region (Erzurum, Kars, Bitlis, Ağrı) and the neighboring Caucasus region. Like other members of the genus, this species is a perennial herbaceous plant characterized by finely divided leaves and compound umbel-shaped inflorescences; these are typical morphological features of the Apiaceae family. Although it shares taxonomic features with the genus *Seseli*, this species mostly prefers high-altitude mountainous areas, rocky slopes and steppes as its habitat. Forming local populations along a line extending from the Erzurum-Kars plateau to Mount Peli in Bitlis, this plant is known locally as “ebem çışır” or “çakşır” (Figure 1) [3,4].

Members of the *Seseli* genus have been used as herbal medicine for many years in traditional medicine for various purposes such as relieving colds, inflammation, pain and gastrointestinal discomfort due to their known therapeutic properties [2]. In addition, it is known that species belonging to this genus have anthelmintic, carminative, stomach-soothing and stimulant properties and are also used in the treatment of central nervous system disorders such as epilepsy [5,6]. In addition to these, some members of the *Seseli* genus are also widely used in traditional medicine due to their antibacterial, antifungal and insect repellent activities [7].

Considering the ethnobotanical uses of species belonging to this genus, various studies have been conducted on their biological activities, and it has been determined that various *Seseli* species exhibit antibacterial, anticancer, anti-inflammatory, and antinociceptive effects [5,8]. In addition, one study showed that *Seseli* species contain essential oils with significant pharmacological potential and these essential oils have antimicrobial and antioxidant properties [9].

Although ethnomedical information about the species *S. transcaucasicum* is limited, various *Seseli* species have long been used in traditional medicine and as food in different regions of Europe and Asia. Depending on the species variety and local ethnobotanical practices, species within this genus have been used to treat colds, gastrointestinal disorders, pain, inflammatory conditions, respiratory diseases, and various infectious diseases. These traditional therapeutic uses are largely attributed to the presence of coumarins, volatile oils, terpenoids, and other bioactive secondary metabolites [2].

One study has shown that *Seseli* species are rich in phenolic compounds (PCs) and possess significant biological activities. In a comprehensive study conducted in Türkiye, it has been reported that the TPC (total phenolic content) of methanol and water extracts of *Seseli gummiferum* and *S. transcaucasicum* species have ranged from 19.09 to 24.33 mg GAE (gallic acid equivalent)/g extract, and the total flavonoid content ranged from 0.45 to 10.09 mg rutin equivalent/g extract. In the same study, it has been reported that the extracts have shown significant antioxidant activity in DPPH (5.51–11.45 mg TE (trolox equivalent)/g), ABTS (43.46–51.91 mg TE/g), CUPRAC (41.67–53.20 mg TE/g) and FRAP (31.26–34.14 mg TE/g) tests; they have also exhibited antibacterial effect against *Staphylococcus lugdunensis* and their cytotoxic activities have been evaluated in HaCaT cells [10].

Studies to date on *Seseli* species and their widespread uses in traditional medicine demonstrate that these plants possess immense healing potential that could benefit human health and be used in the treatment of various diseases. Despite its geographical importance, the species has received limited phytochemical and pharmacological attention compared to other members of the genus [2]. Previous research has shown that *S. transcaucasicum* possesses biologically active metabolites, including phenolic profiles, regular fatty components, and coumarins [10]. Coumarins, particularly pyranocoumarins, have been considered chemotaxonomic markers of the genus, suggesting that *Seseli* species possess antioxidant, anti-inflammatory, antimicrobial, antispasmodic, and other pharmacological activities [1,2]. However, the chemical composition and biological activities of most members of the *Seseli* genus have not yet been sufficiently investigated [2]. A review of studies on *S. transcaucasicum* reveals a lack of comprehensive research, particularly regarding GC-MS, PCs, antimicrobial activity, and cytotoxic activity. This study was conducted to comparatively evaluate the antioxidant, anticancer, and antimicrobial activities of methanol and acetone extracts of *S. transcaucasicum* and to determine the potential effect of the extraction solvent on biological activities. Furthermore, the volatile components of the extracts were identified, their component compositions were characterized, and the biological potential of *S. transcaucasicum* was comprehensively assessed.

## 2. Materials and Methods

### 2.1. Plant Material

**Figure 2:**
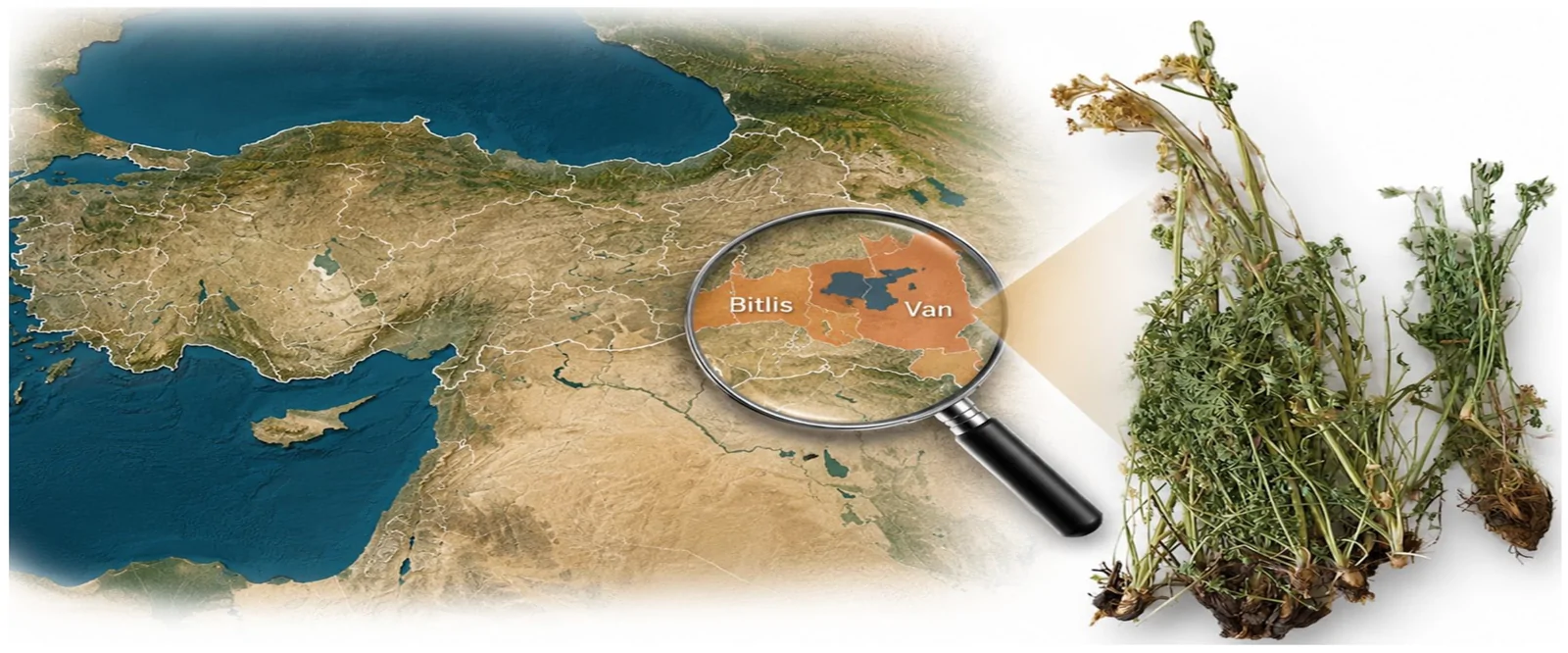
Location where *Seseli transcaucasicum* plant was collected and image of the dried plant.

A complete specimen of the plant *S. transcaucasicum* was collected on 21 June 2023, from the rocky hills of Söğütlü-Evbark villages, located between Bitlis and Van provinces in Türkiye, at an altitude of 2850–2900 m, during its flowering stage (Figure 2). The plant was identified by Prof. Dr. Murat KURSAT and Lutfullah SAKCI and registered as specimen number 8049 in BEUH (Bitlis Eren University Herbarium).

### 2.2. Working Chemicals

All chemicals used were of high purity and were sourced from Merck Millipore (Darmstadt, Germany), Merck EMSURE (Darmstadt, Germany), ISOLAB (Eschau, Germany) and J.T. Baker (Phillipsburg, NJ, USA).

### 2.3. Preparation of the Extract

The collected *S. transcaucasicum* plant was dried without exposure to sunlight, and for this study, the intact flowering parts were selected and ground into a powder in a porcelain mortar. Ten grams of the resulting plant material were weighed and placed in Erlenmeyer flasks, and 100 mL (80% methanol/acetone) was added. The mixture was then incubated at room temperature for 24 h in an orbital shaker at 100 rpm. The resulting solution were then filtered through Whatman No:1 filter paper, and the solvents were removed from the mixture using an evaporator (Hei-VAP Expert/Heidolph, Schwabach, Germany). The resulting was stored at +4 °C until further studies were conducted [10]. Analyses were performed using dissolved extract solutions, and IC_50_ was expressed in this format. However, while the volatile component determination results are based on the raw dry form of the plant material, all other values were calculated based on the dry weight of the crude extracts and reported as mg/g extract.

Traditional solvent extraction was chosen because it has been commonly used in previous phytochemical research on *Seseli* species and allows for direct comparison of phytochemical composition and biological activities with previously published data [10]. Therefore, the same extraction approach was adopted in this study to ensure methodological consistency and facilitate comparison with the existing literature.

Methanol and acetone were selected as extraction solvents because they possess different polarities and are among the most commonly used solvents for recovering phenolic and other bioactive constituents from medicinal plants. Comparing these two solvents enabled evaluation of the effect of solvent polarity on phytochemical composition and biological activities [2,10]. The solvents were completely removed under reduced pressure prior to biological analyses. 

### 2.4. Determination of Volatile Components

Volatile components of *S. transcaucasicum* have been determined using the Head Space-Solid Phase Microextraction (HS-SPME) method on a Shimadzu QP-2020 GC-MS (Kyoto, Japan) device [11]. The flowering parts of the plant wereground and placed in a 2 g SPME vial and were then incubated at 40 °C for 30 min, after which an SPME fiber (Supelco, Bellefonte, PA, USA; 2 cm) was placed inside the vial. The fiber was then allowed to adsorb at the same temperature for 30 min, and after adsorption was complete, it was incubated at 250 °C for 5 min for desorption in the GC-MS injection port. Helium gas, with a flow rate of 1.05 mL/min, was used as the carrier gas, and chromatographic separation was performed using splitless injection mode. The device oven was heated from 40 °C to 240 °C in increments of 4 °C per min and held at this temperature for 6 min. Identification of volatile compounds was performed by comparing mass spectra in the instrument software library (Wiley 9 and NIST11-W9N11-GCMSsolition 4.20 Schimadzu Corporation). RI values of the compounds were calculated according to the method of Dool and Kratz [12] by injecting the n-alkane series (C10–C26) into the GC-MS system under the same chromatographic conditions. Concentrations of the components were calculated based on the peak area of 2.5 µg isobutyl acetate (in methanol) and added as an internal standard to each sample vial (%RSD 5.78).

### 2.5. Determination of Phenolic Compounds

High-performance liquid chromatography (HPLC) was used to determine the PCs of *S. transcaucasicum* [13]. Plant extracts were passed through a 0.45 µm PVDF membrane filter (Millex™, Merck Millipore, Darmstadt, Germany) before starting the study. Then, 20 µL of each sample was taken and injected into a Waters Alliance E2695 HPLC system (Milford, MA, USA). Separation was performed using a reverse-phase C18 column (5 µm, 4.6 × 250 mm; GL Sciences, Tokyo, Japan). Caffeic acid, vanillic acid, *p*-coumaric acid, gallic acid, quercetin, catechin, 3,4-dihydroxbenzoic acid, 4-hydroxbenzoic acid, luteolin, apigenin, and rosmarinic acid solutions were used as standards for determining PCs. Chromatographic data were evaluated using Empower 3 software after measurements were taken with a Photodiode Array (PDA) detector (Waters 2996, Milford, MA, USA) at a wavelength of 280 nm. The amounts of the PCs were calculated in mg/g extract.

### 2.6. Radical Scavenging Activity (RSA)

A method similar to that applied by Osei et al. [14] was used to determine the DPPH (2,2-Diphenyl-1-picrylhydrazyl) scavenging activity of plant extracts, and absorbances were measured using a UV spectrophotometer (Biochrom Libra S70, Cambridge, UK). For this purpose, 2.7 mL of methanolic DPPH solution (60 µM) was added to 0.3 mL of phenolic extract (1 mg/mL, in methanol), and the mixture was left in the dark at room temperature for 30 min At the end of this process, absorbances were measured at 517 nm using a UV-vis spectrophotometer [15]. Trolox was used as a positive control, and each group was examined in three parallels. The DPPH scavenging activity of the extracts was determined as trolox equivalent (TE) by calculating the IC_50_ values from the obtained calibration curves.

### 2.7. Total Phenolic Content Analysis (TPC)

The Folin–Ciocalteu method with a UV spectrophotometer was used to determine the TPC values of the plant extracts [16]. For this purpose, 100 µL of phenolic extract (1 mg/mL, in methanol) was added to a mixture of 900 µL of pure water and 5 mL of Folin–Ciocalteu reagent (0.2 N) and it was left in the dark for 8 min. Then, 5 mL of Na_2_CO_3_ was added to the mixture and vortexed. The absorbance values of the mixture, which was left in the dark for 2 h, were measured at 765 nm using a UV-vis spectrophotometer. TPC was calculated with a calibration curve obtained with different concentrations of gallic acid standard solutions and expressed as gallic acid equivalent (GAE).

### 2.8. Determination of Antimicrobial Activity

The microbial strains used in this study were selected because they are among the most commonly used and clinically relevant reference microorganisms for evaluating the antimicrobial activity of plant extracts. Accordingly, Gram-negative bacterial strains (*Escherichia coli* ATCC 25922 and *Pseudomonas aeruginosa* ATCC 9027), a Gram-positive bacterial strain (*Staphylococcus aureus* ATCC 25923), and a yeast strain (*Candida albicans* ATCC 10231) were used to assess the antimicrobial potential of the tested extracts against a wide range of microorganisms. Besin Broth (Merck, Darmstadt, Germany), Besin Agar (Merck, Darmstadt, Germany), Sabouraud Dextrose Broth (Merck, Darmstadt, Germany), and Sabouraud Dextrose Agar (Merck, Darmstadt, Germany) culture media were used for the cultivation of these strains. Erythromycin 15 µg/disk (Bioanalyse^®^, Ankara, Turkey), Ciprofloxacin 1 µg/disk (Bioanalyse^®^, Ankara, Turkey) and Nystatin 100 µg/disk (Bioanalyse^®^, Ankara, Turkey) disks were used as positive controls, while DMSO (20%) was used as a negative control. Ten microliters of extract prepared by diluting with 20% DMSO (50 and 100 mg/mL) at different concentrations [17] was impregnated into sterile 6 mm diameter empty disks (Bioanalyse, Ankara, Turkey) using the disk diffusion method, and bacterial strains were incubated in an oven at 37 °C for 24 h; yeast strains were incubated at 30 °C for 48 h. After the incubation period was completed, the diameters of the resulting inhibition zones were measured and calculated in mm.

### 2.9. In Vitro Cell Viability Assessment (WST-1 Test)

The HT29 human colorectal adenocarcinoma cell line was used in this study because it is one of the most commonly used and best-characterized in vitro models for evaluating the anticancer potential of natural products against colorectal cancer. HT29 cells were kindly provided by Dr. Mehmet Kadir Erdoğan from the Cancer Research Laboratory of Bingöl University (Bingol, Turkey). The cells were maintained at 37 °C in a humidified atmosphere containing 5% CO_2_ in Dulbecco’s modified Eagle’s medium (DMEM; Sigma-Aldrich, St. Louis, MO, USA; Cat. No. D6429) supplemented with 10% fetal bovine serum (Biowest, Nuaillé, France; Cat. No. S191H), 100 U/mL penicillin, and 100 μg/mL streptomycin (Sigma-Aldrich, St. Louis, MO, USA; Cat. No. P4333).

The cytotoxic effects of the extract were evaluated in mycoplasma-free, exponentially growing HT29 cells using the WST-1 assay, as previously described [18]. Briefly, cells were seeded into 96-well plates (Corning Inc., Corning, NY, USA; Cat. No. 3596) at a density of 5 × 10^3^ cells per well and were allowed to adhere overnight. The following day, the cells were treated with different concentrations of the extract for 72 h Subsequently, 10 μL of WST-1 Cell Proliferation Reagent (Roche Diagnostics GmbH, Mannheim, Germany; 5015944001) was added to each well, and the plates were incubated for an additional 4 h at 37 °C. Absorbance was measured at 450 nm using a Multiskan microplate reader (Thermo Fisher Scientific, Vantaa, Finland; Cat. No. 51119000). Cell viability was expressed as a percentage of the untreated control, which was defined as 100% viability, while 20% DMSO (Sigma-Aldrich, St. Louis, MO, USA; Cat. No. D2650) was included as a positive cytotoxicity control. All experiments were performed using at least three independent biological replicates.

### 2.10. Statistical Analysis

All data obtained in this study were expressed as mean ± standard deviation (SD) values of three independent measurements, and statistical analyses were performed using IBM SPSS Statistics ver. 27 (IBM Corp., Armonk, NY, USA) program. One-way analysis of variance (ANOVA) was used to evaluate differences between groups, and linear regression analysis was applied where appropriate. The statistical significance level was accepted as *p* < 0.05. The results of the GC–MS and HPLC analyses were not included in the statistical analysis because these analyses were performed for the qualitative and quantitative characterization of the extracts, and the reported values represent the measured concentrations of the identified compounds.

## 3. Results and Discussion

### 3.1. Total Phenolic Content and Antioxidant Activity

The total antioxidant capacity (TAC) of *S. transcaucasicum* has been determined using DPPH radical scavenging activity (RSA) and total phenolic content (TPC) methods (Table 1). The R2 value of the total phenolic acid content calculated according to the gallic acid reference was found to be 0.998, and the R2 value of the DPPH scavenging capacity calculated according to the trolox reference was found to be 0.990.

The methanol extract of *S. transcaucasicum* exhibited higher TPC (~77.389 mg GAE/g extract) and RSA (~40.933 mg TE/g extract) values compared to the acetone extract (Table 1). The higher TPC value of the methanol extract is associated with greater radical scavenging activity, indicating a close relationship between TPC and antioxidant activity. In contrast, the acetone extract exhibited lower TPC (~51.932 mg GAE/g extract) and RSA (~16.482 mg TE/g extract) values.

Strong antioxidant properties in plant extracts are generally associated with high TPC. In species belonging to the Apiaceae family, including *S. transcaucasicum*, phenolic acids, flavonoids, and coumarin derivatives are known to exhibit free radical scavenging properties. A recent review study indicated that plants belonging to the Apiaceae family are quite rich in natural antioxidants, and this is largely due to TPC [19].

When the results of this study are examined, similarities to the study by Zengin et al. can be observed [10]—one of the few studies conducted on *S. transcaucasicum*. In this study, methanol and water extracts of *S. transcaucasicum* were compared; it was reported that the methanol extract has a higher total flavonoid content and, in particular, a higher total antioxidant capacity determined by the phosphomolybdenum method compared to the water extract. The researchers explained this situation by stating that solvent polarity has a decisive effect on the amount and composition of the extracted phytochemical compounds. Similarly, although a different solvent system (methanol and acetone) was used in this study, it was determined that the methanol extract had higher TPC and DPPH radical scavenging activity compared to the acetone extract. When both studies are considered together, it is seen that the choice of solvent significantly affects the phenolic composition and antioxidant properties of *S. transcaucasicum.* A recent study on plant species belonging to the Apiaceae family indicate that an increase in the amount of TPC also increases the DPPH radical scavenging capacity [20]. The fact that the methanol extract showed stronger RSA in this study is similar to the other study. In addition to their effects on antioxidant capacity, TPC are known to act as hydrogen donors, neutralizing free radicals and playing an important role in reducing oxidative stress.

In this study, when the IC_50_ values are examined, it was observed that the methanole extract has had a lower IC_50_ value. In the DPPH analysis, a lower IC_50_ value indicates stronger radical scavenging activity. Therefore, this result suggests that the methanole extract may contain some secondary metabolites that are effective against specific radicals. Furthermore, when the TPC and overall antioxidant capacity of this plant are evaluated together, it is seen that the methanol extract has a broader and more comprehensive antioxidant potential due to its richer phenolic profile. In conclusion, it can be said that the *S. transcaucasicum* plant used in this study possesses a significant level of natural antioxidant capacity.

Although the antioxidant potential of *S. transcaucasicum* was demonstrated through in vitro tests, these results may not fully reflect its biological activity under physiological conditions. Therefore, further in vivo studies are needed to evaluate the antioxidant activity, bioavailability, and metabolism of the active components.

### 3.2. Volatile Component Analysis

**Figure 3:**
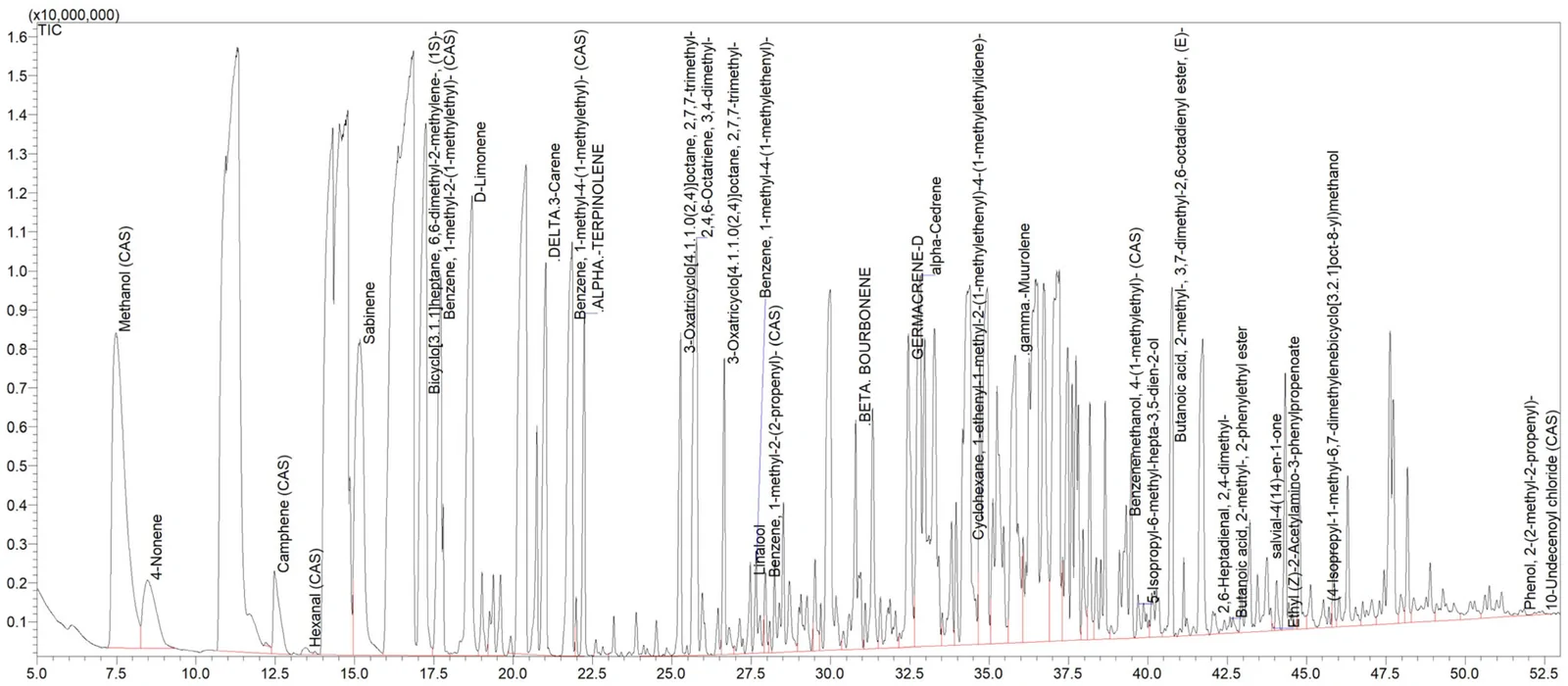
Chromatogram of *Seseli transcaucasicum* obtained by GC-MS.

The volatile components of *S. transcaucasicum* were measured chromatographically, and the resulting chromatogram is given in Figure 3.

**Figure 4:**
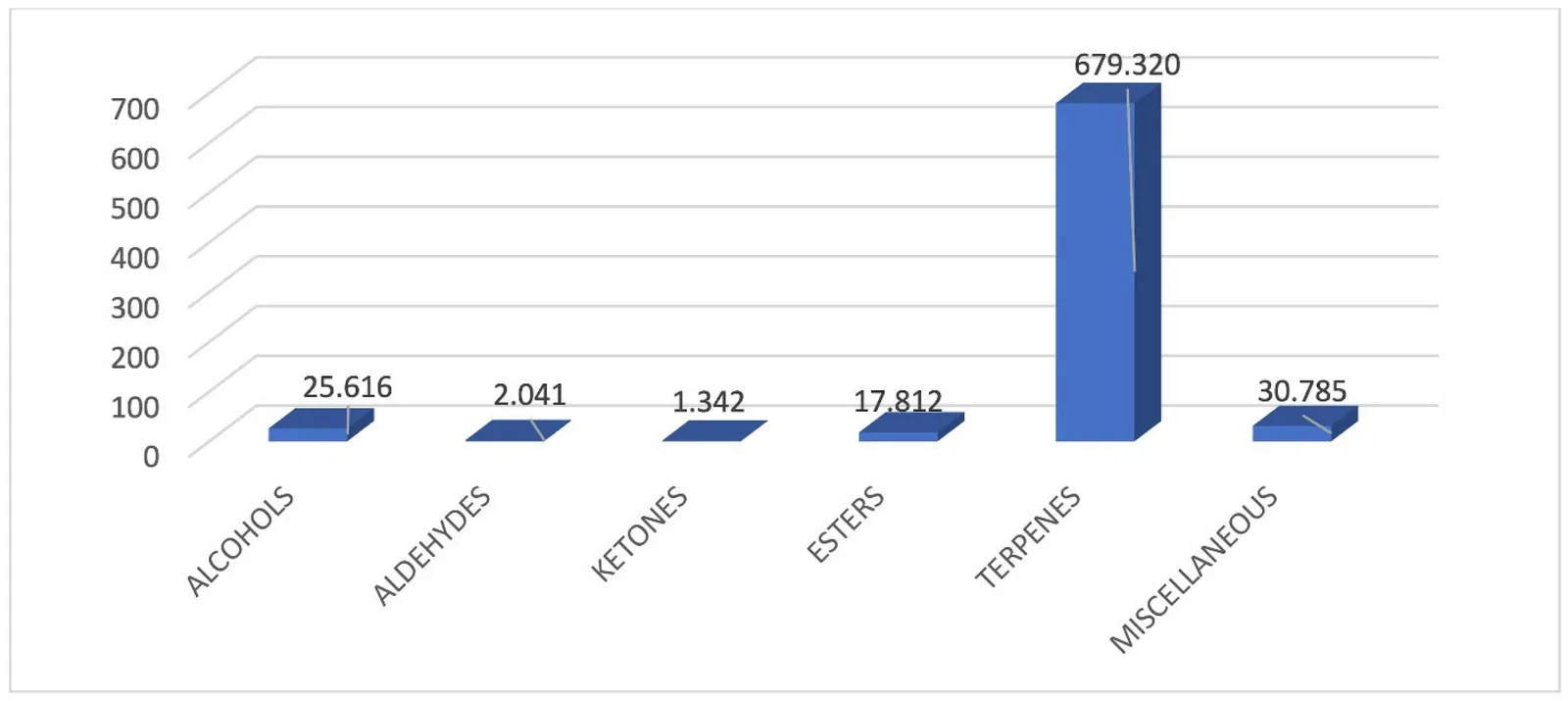
Weight distribution of substance quantities obtained from the GC-MS device of *Seseli transcaucasicum* plant according to the groups in which they are found.

When the flowering parts of *S. transcaucasicum* were examined using a GC-MS instrument, a total of 29 compounds were identified. All peaks in the chromatogram were evaluated in the GC-MS analysis, but only the 29 volatile compounds that can be reliably identified using mass spectra, retention indices (RI), and the NIST mass spectral library have been included in the table. Peaks that cannot be reliably identified, were found in very low abundance, or that cannot be definitively identified due to co-elution have been excluded from the evaluation. These compounds were determined to be 4 alcohols, 2 aldehydes, 1 ketone, 4 esters, 16 terpenes, and 2 miscellaneous compounds (Table 2, Figure 4).

When the volatile components of the flowering parts of *S. transcaucasicum* are examined, it can be seen that the majority of the compounds consist of monoterpene and sesquiterpene terpenoids. When the compounds obtained from this study were examined, the compounds determined in the highest amounts were (-)-β-pinene (220.792 mg/kg), Δ3-carene (81.898 mg/kg), α-cedrene (63.331 mg/kg), elixene (44.535 mg/kg), sabinene (40.822 mg/kg), and D-limonene (38.422 mg/kg). These results show that the flowering parts of *S. transcaucasicum* have a very rich terpenoid content.

When studies on different species of the *Seseli* genus are examined, it can be seen that monoterpene and sesquiterpene compounds are dominant, as was observed in this study. For example, in the study conducted by Janaćković et al. [21], sabinene, α-pinene and β-phellandrene compounds were found in high amounts. When another study was examined, it was observed that the dominant compounds were α-pinene, limonene, camphene and sabinene [9]. These studies, together with the results obtained from this study, show that *Seseli* species generally have a chemical profile rich in terpenoids. This similarity in chemical composition suggests that the volatile profile of *S. transcaucasicum* is consistent with the general chemotaxonomic characteristics of the genus *Seseli*.

In this study, high amounts of monoterpenes such as (-)-β-pinene, sabinene, limonene, and Δ3-carene were detected, which have been shown in previous studies to possess significant biological activities [22]. Of these, β-pinene and limonene exhibit antimicrobial effects by disrupting the permeability of microbial cell membranes, while compounds like sabinene and α-terpinolene enhance antioxidant activity [23]. Furthermore, another study determined that samples rich in germacrene-D, sabinene, and limonene also demonstrated antifungal activity [24]. Therefore, the biological activities observed in the present study may be associated with the combined contribution of these volatile constituents rather than with a single dominant compound.

Although not as abundant as the others, the sesquiterpene compounds obtained in high amounts in this study were germacrene-D, β-bourbonene, γ-muurolene, and α-cedrene. These compounds are also considered to be important in terms of biological activity. Germacrene-D has been shown to possess antimicrobial, anti-inflammatory, and antioxidant properties [25], while sesquiterpenes such as α-cedrene and γ-muurolene have been found to play a role in natural defense mechanisms [26]. Although these compounds were detected at lower concentrations than the major monoterpenes, they may also contribute synergistically to the overall biological properties of the extract.

In addition to the compounds mentioned above, oxygenated compounds such as linalool, geranyl 2-methylbutanoate, and phenethyl 2-methylbutyrate were also detected in this study. Among these, linalool, in particular, has been shown in previous studies to possess antioxidant and antimicrobial properties [27].

This study on *S. transcaucasicum* generally shows agreement with studies on sesquiterpene species and has a chemical profile rich in monoterpenes and sesquiterpenes. In this study, compounds such as (-)-β-pinene, Δ3-carene, sabinene, limonene, and germacrene-D were detected in high amounts. These compounds are thought to be important components affecting the plant′s potential biological activities.

### 3.3. Phenolic Compounds

The PCs profiles of the methanol and acetone extracts of *S. transcaucasicum* were determined by HPLC analysis, and the quantitative results are presented in Table 3.

When the PCs of extracts from *S. transcaucasicum* were examined, it was observed that rosmarinic acid is the compound found in the highest amount in both extracts. Rosmarinic acid was detected at 14.929 mg/g in the acetone extract and 15.195 mg/g in the methanol extract. Rosmarinic acid, known for its strong antioxidant and anti-inflammatory properties—and for being an important derivative of hydroxycinnamic acid—makes a significant contribution to the biological activities of plants [10]. Therefore, it is thought that the high rosmarinic acid content determined in this study plays an important role in the antioxidant potential of the plant extracts. Moreover, the slightly higher concentration of rosmarinic acid in the methanol extract may partially explain its stronger antioxidant performance compared with the acetone extract.

After rosmarinic acid, the compound detected in the highest amount is catechin. The amount of catechin, specifically determined at 14.717 mg/g in the methanol extract, indicates that the extract is rich in flavonoids. Catechins are known to have high free radical scavenging activity, are effective in reducing oxidative stress due to their phenolic structure, and can also exhibit antimicrobial effects against various microorganisms [28,29]. Therefore, the higher catechin content of the methanol extract may also have contributed to its stronger antioxidant and antimicrobial activities observed in the present study.

The presence of flavonoids such as quercetin, apigenin, and luteolin in both extracts, albeit in smaller amounts compared to other compounds, indicates the high phenolic diversity of the plant. Such interactions among phenolic acids may provide broader antioxidant and antimicrobial effects than those expected from individual compounds alone. The higher amount of quercetin in the acetone extract (1.872 mg/g) suggests that solvent selection has an effect on flavonoid extraction. Studies have shown that flavonoids exhibit antioxidant and antimicrobial properties [10].

Although phenolic acids such as caffeic acid, p-coumaric acid, gallic acid, vanillic acid, 3,4-dihydroxybenzoic acid, and 4-hydroxybenzoic acid were detected in lower amounts in this study, they contribute to the overall phenolic profile. The presence of these compounds together suggests that they may synergistically enhance the biological activities of the plant extracts.

A review of the literature reveals that studies on *S. transcaucasicum* have identified chlorogenic acid and narcissin as the main PCs. However, in this study, it is noteworthy that rosmarinic acid and catechin were identified as the dominant compounds among the PCs that studied. The observed differences in compound amounts may be due to variations in the plant′s habitat, climatic conditions, phenological stage, plant part used, and extraction methods. Furthermore, both studies have determined that the species is rich in PCs and possesses significant biological activity potential [10]. These findings indicate that environmental and methodological factors may substantially influence the phenolic composition of *S. transcaucasicum*, even within the same species.

The high concentrations of some of the PCs (rosmanic acid, catechin, quercetin, apigenin) obtained from this study support the findings regarding antimicrobial and antioxidant activity. The presence of these compounds in high amounts suggests that they have an effect on the biological activity of *S. transcaucasicum*. However, it is likely that the biological activities observed in this study result from the combined action of multiple phenolic compounds rather than from the activity of a single constituent.

The phenolic profile of the plant extracts used in the study was determined by HPLC analysis; however, the contribution of individual phenolic compounds to the observed biological activities has not been experimentally confirmed.

### 3.4. Antimicrobial Activity Against Selected Microorganisims

To determine the antimicrobial capacities of extracts obtained from the plant *S. transcaucasicum*, bacterial strains of *E. coli*, *P. aeruginosa*, *S. aureus*, and a yeast strain—*C. albicans*—were used.

The results obtained from methanol and acetone extracts of the flower parts of the *S. transcaucasicum* plant are given in Table 4. When examining the results of this study, it can be seen that the extracts show different levels of inhibition zones against the tested microorganisms. When the differences between the extracts are examined, it can be seen that the methanol extract shows higher activity than the acetone extract. This difference can be explained by the polar nature of methanol and the fact that polar solvents can extract phenolic and terpenoid bioactive compounds more effectively [30]. This observation is also consistent with the higher total phenolic content determined in the methanol extract, suggesting that the greater extraction of phenolic constituents may have contributed to its stronger antimicrobial activity. Among the results of this study, the most effective result was observed in the 100 mg/mL methanol extract of the Gram-positive bacterium *S. aureus* strain, with an inhibition zone of 16 ± 0.471 mm. This indicates that Gram-positive bacteria may be more sensitive to plant extracts. In contrast, the lower inhibition zones obtained against Gram-negative bacteria such as *E. coli* and *P. aeruginosa* may be related to innate resistance mechanisms dependent on the outer membrane structures of these bacteria. Because the outer membrane of Gram-negative bacteria acts as an additional permeability barrier, it can reduce the penetration of bioactive phytochemicals and decrease their antibacterial efficacy.

Previous studies on species belonging to the genus *Seseli* support the antimicrobial results obtained in this study. In a study investigating the antimicrobial effect of volatile compounds obtained from flowering parts, it was determined—as it was in this study—that these compounds showed antimicrobial activity against *E. coli*, *P. aeruginosa*, and *S. aureus* bacteria [21]. Similarly, in another previous study, it was determined that samples obtained from different parts of *S. rigidum* have shown a significant inhibitory effect, especially against *S. aureus* strains [9]. Again, in the same study, monoterpenes such as α-pinene, limonene, camphene, and sabinene have been found in high amounts, as in the volatile compounds obtained in this study. The similarity between the phytochemical profiles reported in previous studies and those obtained in this study may explain the comparable antimicrobial activities observed among *Seseli* species.

It is thought that terpenoid compounds such as (-)-β-pinene, sabinene, limonene, Δ3-carene, and germacrene-D, which were found in high amounts among the volatile components obtained in this study, may contribute to the observed antimicrobial activity. Consistent with this finding, the literature indicates that *Seseli* species rich in monoterpenes exhibit stronger antimicrobial activity, particularly against Gram-positive bacteria [21]. Furthermore, sabinene-rich oils obtained from *S. globiferum* fruits have been reported to exhibit significant antibacterial and antifungal activity [31]. In addition, the antimicrobial activity observed in this study is likely to result from the combined action of multiple volatile and phenolic constituents rather than from the effect of a single compound.

When the antimicrobial effects on *C. albicans*—a yeast strain included in this study—were examined, a low level of effect was observed; however, this result is consistent with previous antifungal activity studies on *Seseli* species [32]. The relatively limited antifungal activity observed against *C. albicans* may indicate that the concentrations of active constituents present in the extracts were more effective against bacterial species than against yeast cells.

In conclusion, the data obtained in this study show that the methanol extracts of *S. transcaucasicum* plant exhibit moderate antimicrobial activity. It is believed that the inhibition zone observed especially on the *S. aureus* strain is due to the high amounts of monoterpene and sesquiterpene compounds among the volatile components. Although the disk diffusion method successfully demonstrated the antimicrobial potential, the lack of MIC data limits the quantitative assessment of the exact therapeutic doses.

### 3.5. Cytotoxicity Study

**Figure 5:**
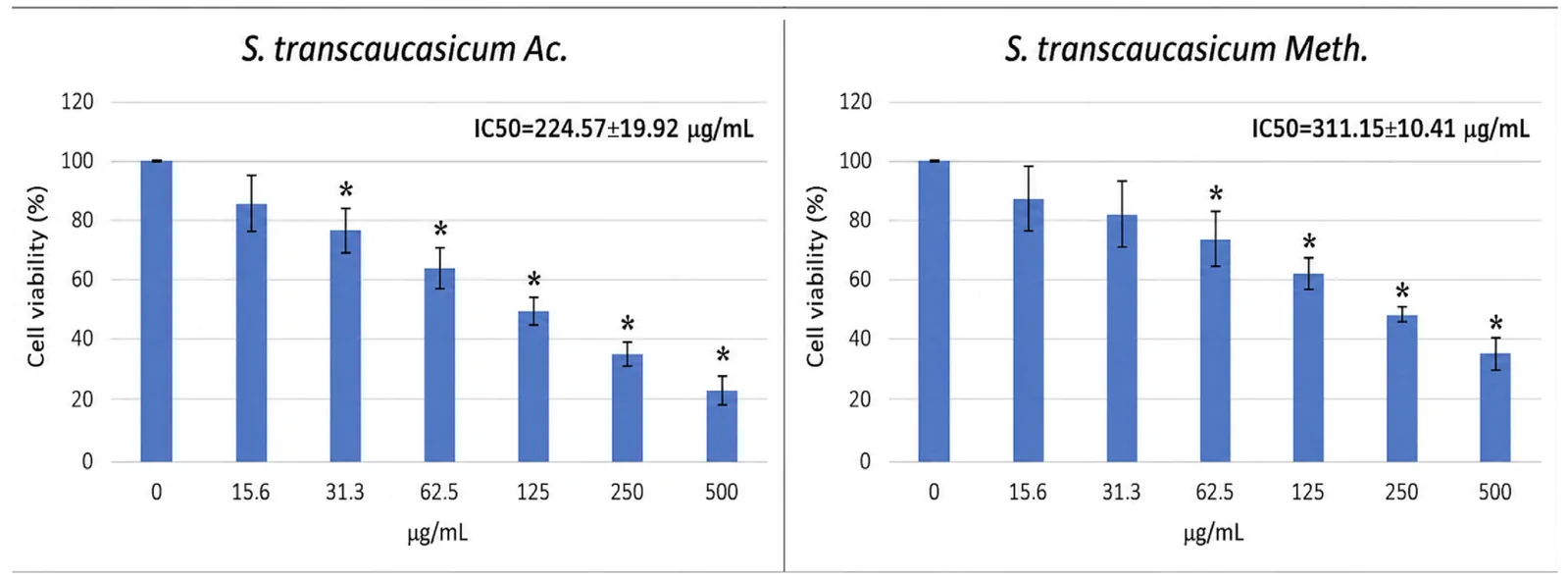
Effects of *Seseli transcaucasicum* extracts on HT29 cell viability and IC_50_ values as determined by the WST-1 assay after 48 h of treatment. Cells were exposed to increasing concentrations of each extract (15.6–500 µg/mL), and cell viability was expressed as a percentage relative to the untreated control group. Data are presented as mean ± standard deviation (SD) from three independent experiments. The “*” symbol indicates a significant difference compared to the control. This difference suggests that the acetone extract has a higher cytotoxicity.

The flowering parts of *S. transcaucasicum* were extracted using methanol and acetone, and their cytotoxic potential against the human colorectal adenocarcinoma cell line HT29 was systematically evaluated using the WST-1 cell viability test. For this purpose, cells were exposed to a wide concentration gradient encompassing seven different dose levels (0–500 µg/mL) during a 48 h incubation period. Cell viability results have been calculated as a percentage compared to a carrier control treated with DMSO (20%) (Figure 5).

At the upper concentration studied—500 µg/mL—the acetone extract reduced viability to 18.03%, while the methanol extract have reduced it to 30.56%. Furthermore, statistically significant difference comparison (IBM-SPSS Statistics ver. 27) has shown that cytosuppressive effect began at 31.3 µg/mL for the acetone sample (*p* = 0.002) and at 62.5 µg/mL for the methanol extract (*p* = 0.012). Considering these values and IC_50_ values together, it can be assumed that the acetone extract has higher cytotoxic activity. This difference suggests that the phytochemical composition of the acetone extract may contain constituents that are more effective in suppressing HT29 cell proliferation, despite its lower total phenolic content compared with the methanol extract.

The most problematic cytotoxic properties of *S. transcaucasicum* are likely due to its PCs. However, the synergistic effects of terpenes, which are present in significant amounts among its volatile components, can also be mentioned. Rosmarinic acid, detected in the highest amounts in both the acetone and methanol extracts (14.93–15.20 mg/g ext., respectively), is a compound whose antioxidant and cytotoxic properties have been highlighted in previous studies [33,34]. Furthermore, catechin, another important PC, was detected in higher amounts—particularly in the methanol extract (14.72 mg/g ext.)—compared to the acetone extract (3.31 mg/g ext.). Catechin is a biomolecule with antitumor effects, particularly acting on apoptosis and signaling pathways [35].

In the acetone extract, the PCs measured to be significantly higher than in the methanol extract were quercetin (1.87 mg/g in A.ext.—1.01 mg/g in M.ext.) and apigenin (1.26 mg/g in A.ext.—0.24 mg/g in M.ext.). Apigenin was previously reported to exhibit cytotoxic effects in many cancer cell lines such as breast, colon, prostate, lung, melanoma, and osteosarcoma through apoptosis induction, cell cycle arrest, and suppression of signaling pathways [36], and also to synergize with chemotherapeutic drugs [37]. Similarly, quercetin can exert antiproliferative effects through mechanisms such as mitochondrial apoptosis activation, ROS modulation, and DNA damage [38]. The higher presence of such important PCs may play a significant role in the acetone extract exhibiting a higher cytotoxic effect compared to the methanol extract. These findings also indicate that qualitative differences in the phenolic profile, rather than only the total phenolic content, may influence the cytotoxic response of the extracts.

While volatile compounds are not as effective as phenols in cytotoxic activity, they have been reported to induce early and late apoptosis in cancer cells, depolarizing the mitochondrial membrane and leading to various morphological changes and shifts in apoptotic proteins [39]. For example, significant amounts of the main component (-)-β-Pinene (220.79 mg/kg DW) were detected in GCMS analysis, and previous studies have reported that monoterpenes can exert cytotoxic effects through inducing apoptosis, increasing oxidative stress, and suppressing the cell cycle [40,41]. Another major compound, Δ3-Carene (81.90 mg/kg DW), exhibits cytotoxic effects by disrupting cell membrane integrity, modulating oxidative stress, causing mitochondrial dysfunction, and triggering apoptotic mechanisms [41,42]. D-Limone (38.42 mg/kg DW) is another dominant compound proven to have anticancer effects on colon cancer (Caco-2) [43].

In a previous study, the cytotoxic effects of the same plant on the HaCaT cell line have been investigated, and the IC_50_ has been reported as 321.41 ± 1.25 µg/mL for the water extract and >500 µg/mL for the methanol extract [10]. The authors have noted that the water extracts were more toxic than the methanol extracts and argued that this effect might be related to the phenolic profile.

The cytotoxicity of the samples on HT29 cells within the tested concentration range is likely related to their unique phytochemical composition. Rosmarinic acid, catechin, quercetin, and apigenin, detected by HPLC analysis, and the aforementioned components, along with high amounts of (-)-β-Pinene, Δ3-Carene, and D-Limone, detected by GC-MS analysis, suggest an effect on anticancer activity; however, their individual antiproliferative effect is considered insufficient. This supports the view that cytotoxic activity is driven not by a single compound, but by the synergistic interaction of multiple components within the overall phytochemical matrix. While cytotoxicity was effectively demonstrated on cancerous cell lines, the absence of a non-cancerous (healthy) cell line as a control limits our understanding of the selectivity index.

In this study, cytotoxic activity was evaluated only against the HT29 colorectal adenocarcinoma cell line. Further studies using additional cancer cell lines and normal human cells to determine the selectivity and therapeutic potential of the extracts will reveal the cytotoxic activity of this plant more comprehensively.

## 4. Conclusions

This study details the phytochemical content and antioxidant, antimicrobial, and cytotoxic activities of *S. transcaucasicum* extracted using methanol and acetone, a topic that has not been sufficiently researched before. The results show that this plant is a rich source of natural bioactive compounds with significant biological activities. Antioxidant analysis results show that the methanol extract has a higher TPC and a higher free radical scavenging capacity compared to the acetone extract. Both extracts of *S. transcaucasicum* are particularly rich in PCs, especially rosmarinic acid, catechin, and quercetin. The volatile components of *S. transcaucasicum* are also rich in terpene compounds such as β-pinene, Δ3-carotene, α-cedrene, elixene, sabinene, and D-limonene. It has been observed that *S. transcaucasicum* forms varying levels of inhibitory zones on Gram-positive and Gram-negative bacteria and yeast strains, and also exhibits a dose-dependent antiproliferative effect on the HT29 human colorectal adenocarcinoma cell line.

This study differs from previous studies on this plant, particularly in the identification of volatile components, the identification of PCs, the determination of its antimicrobial effect using the disk diffusion method, and the determination of its cytotoxic effects on the HT29 human colorectal adenocarcinoma cell line. When the results of the study are evaluated as a whole, it can be concluded that *S. transcaucasicum* possesses strong antioxidant properties, a high PCs content, and biological activity, and is a plant species rich in terpenic compounds. These findings demonstrate that the plant is an important phytotherapy resource that can be evaluated in the development of natural antioxidant, antimicrobial, and potential anticancer agents. Future studies will contribute to a more comprehensive understanding of the plant′s pharmacological potential by focusing on isolating the responsible active compounds, elucidating their mechanisms of action, and validating their efficacy in in vivo models.

Overall, this study presents the first comprehensive assessment of the phytochemical composition and biological activities of *S. transcaucasicum*. However, future research, including isolation based on biological activity, mechanistic studies, animal models, toxicity assessments, and clinical trials, is needed to validate its potential applications in pharmaceutical and nutraceutical fields.

## Figures and Tables

**Table 1:** Total phenolic content and antioxidant activity of *Seseli transcaucasicum* extracts.

Samples	TPC (mg GAE/g Ext.)	RSA (mg TE/g Ext.)	IC_50_ (mg/L)
*S. transcaucasicum* M	77.389 ± 1.389	40.933 ± 0.067	25.115 ± 0.762
*S. transcaucasicum* A	51.932 ± 0.926 *	16.482 ± 0.151 *	34.670 ± 0.780 *

M: Methanol, A: Acetone, TPC: Total phenolic content, RSA: Radical scavenging activity, IC_50_: Half maximal inhibitory concentration, TE: Trolox equivalent, GAE: Gallic acid equivalent, Ext.: Extract. “*” indicates a significant difference between the number in the same column and the other number (n = 3, *p* < 0.001, ANOVA), and shows that the results of these two analyses are very different from each other.

**Table 2:** Volatile components and their classes obtained from *Seseli transcaucasicum* plant using GC-MS instrument.

Compound Name	RT	RI	Amount (mg/kg DW)
ALCOHOLS			
Linalool	27.490	1414	3.191
Cuminyl acetate	39.330	1825	16.730
5-Isopropyl-6-methyl-hepta-3,5-dien-2-ol	39.715	1841	3.611
Abietyl alcohol	45.550	2095	2.084
ALDEHYDES			
Hexanal	13.470	1108	0.699
2,6-Heptadienal, 2,4-dimethyl-	42.110	1943	1.342
KETONES			
Salvialenone	43.770	2017	13.512
ESTERS			
Geranyl 2-methylbutanoate	40.765	1885	15.300
Phenethyl 2-methylbutyrate	42.605	1965	1.897
Ethyl (Z)-2-Acetylamino-3-phenylpropenoate	43.950	2024	0.185
10-Undecenoyl chloride	52.445	2400	0.430
TERPENES			
(-)-β-Pinene	17.235	1180	220.792
α-Pinene oxide	25.285	1356	22.882
Camphene	12.490	1087	7.837
Sabinene	15.180	1141	40.822
D-Limonene	18.685	1208	38.422
Δ3-Carene	21.030	1258	81.898
α-Terpinolene	22.250	1283	13.140
4-Nonene	8.470	989	13.141
*o*-Cymene	17.715	1189	30.617
*p*-Cymenene	21.835	1274	36.575
*o*-Allyltoluene	27.960	1427	2.524
β-Bourbonene	30.805	1512	14.275
Germacrene-D	32.475	1568	20.012
α-Cedrene	32.830	1580	63.331
Elixene	34.375	1634	44.535
γ-Muurolene	35.825	1687	28.517
MISCELLANEOUS			
3,4-Dimethyl-2,4,6-octatriene	25.780	1369	30.131
6-Allyl-2-cresol	51.765	2370	0.654

RT: Retention Time (min), RI: Retention Index, Amount: mg/kg DW plant.

**Table 3:** The amounts of phenolic compounds in *Seseli transcaucasicum* plant (mg/g extract).

Phenolic Compounds	*S. transcaucasicum* A	*S. transcaucasicum* M
Caffeic acid	0.040	0.044
Vanilic acid	0.024	0.044
*p*-Coumaric acid	0.052	0.038
Gallic acid	0.930	0.606
Quercetin	1.872	1.010
Catechin	3.310	14.717
3,4-Dihydroxybenzoic acid	0.384	0.710
4-Hydroxybenzoic acid	0.024	0.144
Luteolin	0.29	0.28
Apigenin	1.263	0.239
Rosmarinic acid	14.929	15.195

M: Methanol, A: Acetone.

**Table 4:** Antimicrobial activity of *Seseli transcaucasicum* methanol and acetone extracts against selected microorganisms.

Strains	Antibiotic (PC)	Results (mm)	Methanol Extract (mm)		Acetone Extract (mm)	
			100 mg/mL	50 mg/mL	100 mg/mL	50 mg/mL
*Escherichia coli*	1 µg Ciprofloxacin/disk	25.0 ± 0.5	12.0 ± 0.5 *	11.0 ± 0.0 *	11.0 ± 0.4	10.0 ± 0.5 *
*Staphylococcus aureus*	15 µg Erythromycin/disk	26.0 ± 0.9	16.0 ± 0.5 *^,a^	11.0 ± 0.5 *^,a^	8.0 ± 0.0 *	8.0 ± 0.5 ^a^
*Candida* *albicans*	100 µg Nystatin/disk	28.0 ± 1.3	9.0 ± 0.5	8.0 ± 0.5 *	9.0 ± 0.0 *	-
*Pseudomonas aeruginosa*	10 µg Gentamisin/disk	18.0 ± 3.8	9.0 ± 0.5	8.0 ± 0.5 *	10.0 ± 0.5 *	9.0 ± 0.4

A significant difference was found between values containing the same symbol in exponential notation within a single row (ANOVA, Tukey, n = 3, *p* < 0.05). The symbol “*” indicates a significantly different group of extracts compared to a positive control (antibiotic/antifungal), while the symbol “^a^” indicates a difference in resistance between different extract concentrations or extract types for the same organism. PC: Positive control. -: Used to indicate that it showed no effect.

## Data Availability

Data will be made available on request. The data that support the findings of this study are available from the corresponding author upon reasonable request.

## References

[1] Tosun A. (2003). Chemical constituents and biological activity of *Seseli* L. (Umbelliferae) species. J. Fac. Pharm. Ank. Univ..

[2] Onder A., Nahar L., Cinar A.S., Sarker S.D. (2023). The genus *Seseli* L.: A comprehensive review on traditional uses, phytochemistry, and pharmacological properties. J. Herb. Med..

[3] Güner E.D., Duman H. (2013). The revision of genus *Seseli* (Umbelliferae) in Turkey. Turk. J. Bot..

[4] Menglan S., Fading P., Zehui P., Watson M.F., Cannon J.F., Holmes-Smith I., Kljuykov E.V., Phillippe L.R., Pimenov M.G. (2005). Apiaceae (Umbelliferae). Flora China.

[5] Küpeli E., Tosun A., Yesilada E. (2006). Anti-inflammatory and antinociceptive activities of *Seseli* L. species (Apiaceae) growing in Turkey. J. Ethnopharmacol..

[6] Sahranavard S., Ghafari S., Mosaddegh M. (2014). Medicinal plants used in Iranian traditional medicine to treat epilepsy. Seizure.

[7] Ilić M.D., Jovanović V.P.S., Mitić V.D., Jovanović O.P., Mihajilov-Krstev T.M., Marković M.S., Stojanović G.S. (2015). Comparison of chemical composition and biological activities of *Seseli rigidum* fruit essential oils from Serbia. Open Chem..

[8] Cinar A.S., Bakar-Ates F., Onder A. (2020). *Seseli petraeum* M. Bieb. (Apiaceae) Significantly inhibited cellular growth of A549 lung cancer cells through G0/G1 cell cycle arrest. An. Acad. Bras. Ciênc..

[9] Marčetić M., Božić D., Milenković M., Lakušić B., Kovačević N. (2012). Chemical composition and antimicrobial activity of essential oil of different parts of *Seseli rigidum*. Nat. Prod. Commun..

[10] Zengin G., Stojković D., Mahomoodally M.F., Jugreet B.S., Paksoy M.Y., Ivanov M., Gašić U., Gallo M., Montesano D. (2021). Comprehensive Biological and Chemical Evaluation of Two *Seseli* Species (*S*. *gummiferum* and *S. transcaucasicum*). Antioxidants.

[11] Genovese C., Timpanaro N., Nicolosi D., Tempera G. (2015). Chemical composition of *Centaurea* extracts. Pharmacogn. Res..

[12] Van Den Dool H.A.N.D., Kratz P.D. (1963). A generalization of the retention index system including linear temperature programmed gas-liquid partition chromatography. J. Chromatogr. A.

[13] Veneziani R.C.S., Ambrósio S.R., Martins C.H.G., Lemes D.C., Veneziani L.F. (2018). Chemical composition, antimicrobial and antioxidant activities of essential oils from *Centaurea* species. Ind. Crops Prod..

[14] Osei J.B., Amiri A., Wang J., Tavares M.T., Kiatkittipong W., Najdanovic-Visak V. (2022). Recovery of oils and antioxidants from olive stones. Biomass Bioenergy.

[15] Tawaha K., Alali F.Q., Gharaibeh M., Mohammad M., El-Elimat T. (2007). Antioxidant activity and total phenolic content of selected Jordanian plant species. Food Chem..

[16] Capanoglu E., De Vos R.C., Hall R.D., Boyacioglu D., Beekwilder J. (2013). Changes in polyphenol content during production of grape juice concentrate. Food Chem..

[17] NCCLS (1997). Performance Standards for Antimicrobial Disk Susceptibility Tests.

[18] Graham E., Rampazzo L., Leung C.W.B., Wall J., Gerőcz E.Z., Liskovykh M., Goncharov N., Saayman X., Gundogdu R., Kanemaki M.T. (2025). The homologous recombination factors BRCA2 and PALB2 interplay with mismatch repair pathways to maintain centromere stability and cell viability. Cell Rep..

[19] Jayakodi Y., Thiviya P., Gamage A., Evon P., Madhujith T., Merah O. (2024). Antioxidant activity of essential oils extracted from Apiaceae family plants. Agrochemicals.

[20] Ulewicz-Magulska B., Wesolowski M. (2023). Antioxidant activity of medicinal herbs and spices from plants of the Lamiaceae, Apiaceae and Asteraceae families: Chemometric interpretation of the data. Antioxidants.

[21] Janaćković P., Soković M., Vujisić L., Vajs V., Vucković I., Krivošej Z., Marin P.D. (2011). Composition and antimicrobial activity of *Seseli globiferum* essential oil. Nat. Prod. Commun..

[22] Melkina O.E., Plyuta V.A., Khmel I.A., Zavilgelsky G.B. (2021). The mode of action of cyclic monoterpenes (−)-limonene and (+)-α-pinene on bacterial cells. Biomolecules.

[23] Zielińska-Błajet M., Feder-Kubis J. (2020). Monoterpenes and their derivatives—Recent development in biological and medical applications. Int. J. Mol. Sci..

[24] Milosavljević S., Tešević V., Vučković I., Jadranin M., Vajs V., Soković M., Janaćković P., Jovanović A. (2007). Composition and antifungal activity of the essential oil of *Seseli annuum* wild-growing in Serbia. Fitoterapia.

[25] De Cássia Da Silveira e Sá R., Andrade L.N., De Sousa D.P. (2015). Sesquiterpenes from essential oils and anti-inflammatory activity. Nat. Prod. Commun..

[26] Lesjak M.M., Beara I.N., Orčić D.Z., Petar K.N., Simin N.Đ., Emilija S.Đ., Mimica-Dukić N.M. (2014). Phytochemical composition and antioxidant, anti-inflammatory and antimicrobial activities of Juniperus macrocarpa Sibth. et Sm. J. Funct. Foods.

[27] Mączka W., Duda-Madej A., Grabarczyk M., Wińska K. (2022). Natural compounds in the battle against microorganisms—Linalool. Molecules.

[28] Bernatoniene J., Kopustinskiene D.M. (2018). The role of catechins in cellular responses to oxidative stress. Molecules.

[29] Taylor P.W., Hamilton-Miller J.M., Stapleton P.D. (2005). Antimicrobial properties of green tea catechins. Food Sci. Technol. Bull..

[30] Lee J.E., Jayakody J.T.M., Kim J.I., Jeong J.W., Choi K.M., Kim T.S., Seo C., Azimi I., Hyun J., Ryu B. (2024). The influence of solvent choice on the extraction of bioactive compounds from Asteraceae: A comparative review. Foods.

[31] Stojković D., Glamočlija J., Soković M., Grubišić D., Petrović S., Kukić J., Ristić M. (2008). Chemical composition, antimicrobial and antiradical properties of the essential oils of *Seseli globiferum* fruits. Nat. Prod. Commun..

[32] Matejić J.S., Džamić A.M., Mihajilov-Krstev T., Ranđelović V.N., Krivošej Z.Đ., Marin P.D. (2012). Total phenolic content, flavonoid concentration, antioxidant and antimicrobial activity of methanol extracts from three *Seseli* L. taxa. Cent. Eur. J. Biol..

[33] Matejczyk M., Świsłocka R., Golonko A., Lewandowski W., Hawrylik E. (2018). Cytotoxic, genotoxic and antimicrobial activity of caffeic and rosmarinic acids and their lithium, sodium and potassium salts as potential anticancer compounds. Adv. Med. Sci..

[34] Huerta-Madronal M., Caro-Leon J., Espinosa-Cano E., Aguilar M.R., Vazquez-Lasa B. (2021). Chitosan–Rosmarinic acid conjugates with antioxidant, anti-inflammatory and photoprotective properties. Carbohydr. Polym..

[35] Cheng Z., Zhang Z., Han Y., Wang J., Wang Y., Chen X., Shao Y., Cheng Y., Zhou W., Lu X. (2020). A review on anti-cancer effect of green tea catechins. J. Funct. Foods.

[36] Yan X., Qi M., Li P., Zhan Y., Shao H. (2017). Apigenin in cancer therapy: Anti-cancer effects and mechanisms of action. Cell Biosci..

[37] Nozhat Z., Heydarzadeh S., Memariani Z., Ahmadi A. (2021). Chemoprotective and chemosensitizing effects of apigenin on cancer therapy. Cancer Cell Int..

[38] Sakao K., Hamamoto S., Urakawa D., He Z., Hou D.X. (2024). Anticancer activity and molecular mechanisms of acetylated and methylated quercetin in human breast cancer cells. Molecules.

[39] Delgado C., Mendez-Callejas G., Celis C. (2021). Caryophyllene oxide, the active compound isolated from leaves of *Hymenaea courbaril* L. (Fabaceae) with antiproliferative and apoptotic effects on PC-3 androgen-independent prostate cancer cell line. Molecules.

[40] Hou J., Zhang Y., Zhu Y., Zhou B., Ren C., Liang S., Guo Y. (2019). α-Pinene induces apoptotic cell death via caspase activation in human ovarian cancer cells. Med. Sci. Monit. Int. Med. J. Exp. Clin. Res..

[41] Doytchinova I. (2022). Drug Design—Past, Present, Future. Molecules.

[42] Perumalsamy H., Sukweenadhi J., Ranjan A., Dubey A., Mahadev M., Elsadek M.F., Almutairi S.M., Sohn D., Balusamy S.R. (2025). Structural isomers of carene persuade apoptotic cell death by inhibiting cell cycle in breast cancer cells: An in silico and in vitro approach. Tissue Cell.

[43] Alghamdi A.A. (2025). D-limonene exhibits antiproliferative activity against human colorectal adenocarcinoma (Caco-2) cells via regulation of inflammatory and apoptotic pathways. Curr. Issues Mol. Biol..

